# Spatiotemporal Characteristics and Driving Force of Ecosystem Health in an Important Ecological Function Region in China

**DOI:** 10.3390/ijerph17145075

**Published:** 2020-07-14

**Authors:** Wei Shen, Zhicheng Zheng, Yaochen Qin, Yang Li

**Affiliations:** 1 College of Environment and Planning, Henan University, Kaifeng 475004, China; shenwei@henu.edu.cn (W.S.); zhengzhicheng@henu.edu.cn (Z.Z.); liyang.rs@henu.edu.cn (Y.L.); 2 Key Laboratory of Geospatial Technology for Middle and Lower Yellow River Region, Henan University, Kaifeng 475004, China

**Keywords:** ecosystem health, driving force, Geodetector model, affected area, Yellow River

## Abstract

Quantitative assessment can scientifically determine the health status of a regional ecosystem, identify regional eco-environmental problems, and assist in promoting regional sustainable development and environmental management. Taking China’s important ecological function region, the Yellow River affected area as an example, this study constructed an extended evaluation index system based on the pressure-state-response framework, and remote sensing and GIS techniques were used to dynamically evaluate the spatial and temporal characteristics of ecosystem health in the study area. Furthermore, influencing factors on ecosystem health in the study area were extensively analyzed using the GeoDetector model. The results show that the ecosystem health level in the study area shows significant spatial heterogeneity from 1995–2015, and showed a fluctuating change process. Areas with large fluctuations in health level were mainly distributed in extreme climate areas, ecologically fragile areas, on plains and in hilly areas. Spatial differences of ecosystem health were well explained by using the biological abundance index, relief degree of land surface, soil type, annual average precipitation, elevation, annual average temperature, and population density. Influencing factors have significant interactive effects on ecosystem health.

## 1. Introduction

Damage to the ecological environment is a major global issue that needs to be studied and resolved [1,2]. However, with an increase in the scope and intensity of human social and economic activities, human disturbance has produced a series of significant negative problems on different regional scales related to land cover, biodiversity, hydrological systems and local climate [1,3,4], such as ecosystem degradation, environmental pollution and global warming [5,6,7]. At the same time, the ecological environment limits human social and economic development to a certain extent, resulting in a gradual realization that the maintenance of ecosystem health is very important. For the majority of developing countries experiencing rapid economic development, such as China, India and Brazil, problems associated with environmental pollution have become prominent, so eco-environmental problems have been paid close attention to and the governments of these countries are committed to improvement. Ecosystem health is an important factor for regional sustainable development. The study of ecosystem health is helpful in understanding the health status of regional ecosystems and identifying eco-environmental problems and plays an important role in promoting regional sustainable development and environmental management.

With the deepening of regional sustainable development research, ecosystem health as a new goal of environmental management and sustainable development has become a hot spot and trend in ecological environment research. The majority of initial studies of ecosystem health have focused on conceptual definitions and emerging problem reviews [8,9,10,11]. Ecosystem health refers to the environment’s capacity for self-organization, self-maintenance, and recovery from stress on a temporal scale [11]. As healthy ecosystems can provide the material basis and ecological services for human survival, maintaining healthy ecosystems is fundamental to guarantee the achievement of regional sustainable development [12]. A regional ecosystem is a huge and complex system composed of different types of ecosystems, a network with multi-dimensional interactions [13]. Therefore, it is necessary to establish a comprehensive assessment model to evaluate ecosystem health. Evaluation frameworks include the “pressure-state-response” (PSR) framework [14,15,16], the “vitality-organization-resilience” (VOR) evaluation framework [17,18,19,20], the subsystem model, and the natural-social-economic model [21]. Among these, the PSR evaluation framework takes into account the structural and functional integrity of the ecosystem, as well as the level of resilience, stress and response [15,16]. The VOR evaluation framework places emphasis on the structural and functional integrity of the ecosystem, as well as landscape pattern changes, but ignores stress and response levels under the influence of human activities [15]. The subsystem model and natural-social-economic model ignore land use/land cover change and related landscape pattern change [14,16]. A healthy ecosystem should not only have the ability to provide ecological services for human beings, but should also have the ability to maintain its own sustainable development [20]. The state of ecosystem health is the result of long-term interactions between natural, social and economic factors, having comprehensive, complex and systematic characteristics. Therefore, the evaluation framework should reflect not only the structure and function of the ecosystem, but also the impact of human activities on ecosystem health. The PSR evaluation framework can comprehensively reflect the ecosystem health of regions which contain more human activities [15,16]. Based on this, we have selected the “pressure-state-response” (PSR) evaluation framework to organize evaluation indicators, so as to comprehensively evaluate the health status of the ecosystem in the research area.

In general, more attention has been paid recently to ecosystem health. However, the study of ecosystem health mainly focuses on the application of evaluation models and methods, as well as analysis and evaluation of empirical research in various typical areas [22,23,24,25,26,27,28]. However, from the existing research, there are relatively few studies on the influencing factors on ecosystem health. Traditional methods such as the regression model [29], correlation analysis [30,31] and principal component analysis [22] are usually used for this. However, traditional statistical analyses or spatial analysis methods cannot quantify the interactions of the driving factors and their combining effects on ecosystem health that are induced by the complexity of geographic processes [32,33]. Compared with the traditional analysis methods, the Geodetector model is a more comprehensive statistical method [33]. It can not only analyze the relative importance (or explanatory power) of influencing factors, but also analyze the interaction of multiple factors on ecosystem health, which is lacking in other studies. Based on this, this paper uses the Geodetector model.

The Yellow River affected area refers to the largest geographical area affected by the Yellow River basin, the Yellow River flood disaster and Yellow River agricultural irrigation. The region plays an important role in protecting, restoring, and improving regional water conservation, preventing wind and fixing sand, conserving water and soil, storing flood water and protecting biodiversity, and is an important ecological function region in China. However, in recent years, the rapid economic development and the rapid expansion of urbanization have caused obvious changes in regional land use patterns, ecological landscape patterns and the atmospheric water environment. Therefore, the main objective of this paper is, taking The Yellow River affected area as an example, to identify a proper assessment method and the corresponding appropriate indicators to assess the ecosystem health conditions of the study area, and to analyze the influencing factors of ecosystem health spatial differentiation. It is of great significance to understand the regional ecosystem health status, identify eco-environmental problems, and improve regional sustainable development and environmental management.

## 2. Study Area and Data Source

### 2.1. Study Area

**Figure 1:**
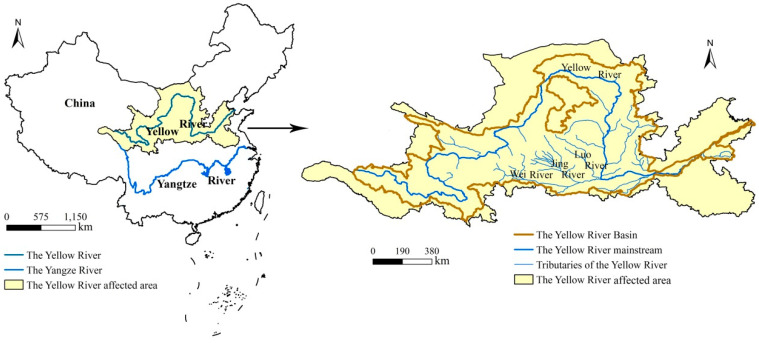
The location of the Yellow River affected area in China and an overview of the study area.

The Yellow River affected area spans 13 provinces, special municipalities (directly managed by the Central Government) in eastern, central and western China, including 531 cities and counties. The Yellow River affected area has a total area of 1,412,900 km^2^, including 752,000 km^2^ (Figure 1) of the natural basin. From the west to the east this area spans four geomorphological units (the Qinghai-Tibet Plateau, the Inner Mongolia Plateau, the Loess Plateau and the Huang-Huai-Hai Plain), and it includes a variety of climate types and different levels of social and economic development. This area is one of the most densely populated areas in the world, having extremely intensive human social and economic activities. Since the reform and opening up of China, the rapid development of industry and the rapid expansion of land urbanization have resulted in significant changes of regional landscape patterns, and air and water quality. As such, this region is a typical case area through which to study ecosystem health evolution in the last 20 years.

### 2.2. Data Sources

In our study, the vector boundary data of the Yellow River affected area was derived from the National Science and Technology basic condition platform of the China-National Earth system Science data Center (http://www.geodata.cn)). The scale of the study area is 1:2,000,000. Research data include land use raster data, the normalized difference vegetation index (NDVI) spatial distribution data set, vegetation net primary productivity (NPP), temperature, precipitation, geomorphological and geological data, GDP, and population spatial distribution kilometer grid data (Table 1). The research data set was provided by the Data Center for Resources and Environmental Sciences, Chinese Academy of Sciences (RESDC) (http://www.resdc.cn) and the Geographical Information Monitoring Cloud Platform (http://www.dsac.cn).

## 3. Methodology

### 3.1. Framework of Ecosystem Health Assessment

A healthy ecosystem should not only have the ability to promote stable ecological services for human beings, but also have the capacity for sustainable development to maintain the integrity of its structure and function [20,34,35]. The PSR evaluation framework takes into account the structural and functional integrity of the ecosystem, as well as its level of resilience, stress and response of [14,15,16], so it can comprehensively reflect the ecosystem health state under the influence of human activities. Based on this, this study selected the “pressure-state-response” (PSR) evaluation framework model to organize evaluation indicators. In this model, the pressure layer describes the pressure caused by human activities in the ecosystem, and clarifies the degree of pressure on the ecosystem, including resource pressure and population pressure. The state layer describes the comprehensive development state of the ecosystem, including the vitality, organization, and resilience of the ecosystem. Among these, vitality represents the level of ecosystem vitality, organization characterizes the structural stability of the ecosystem, and elastic force characterizes the ability of the ecosystem to resist external interference and restore its original state after destruction. The response layer describes the degree of response of the ecosystem to external disturbances, including changes in natural ecosystems and human activities.

### 3.2. Ecosystem Health Evaluation

#### 3.2.1. Evaluation Unit

The size of the evaluation unit was determined based on pixel resolution, study area, and index calculation [28,36,37]. In our paper, the pixel resolution of the remote sensing data was 1 km × 1 km; the study area was approximately 1,412,900 km^2^ in 2015; calculations of some indicators, such as the biological abundance index, the ecological elasticity index, relief degree of land surface, and human interference, demanded a sufficient area for the evaluation unit. The unit size should satisfy the spatial heterogeneity demand, contain sufficient areas and pixel numbers, and avoid excessive data. If the size was too small, then the unit could not meet the area demand of the indicator calculation and would create unnecessary data; if the size was too large, then it could not exhibit spatial heterogeneity. Therefore, referring to relevant research [28,36,37], the 8 km × 8 km grids were generated using the Create Fishnet tool in ArcGIS 10.3, which could balance the aforementioned demands. Finally, the number of evaluation units in the research area were 19,779.

#### 3.2.2. Construction of the Index System

**Figure 2:**
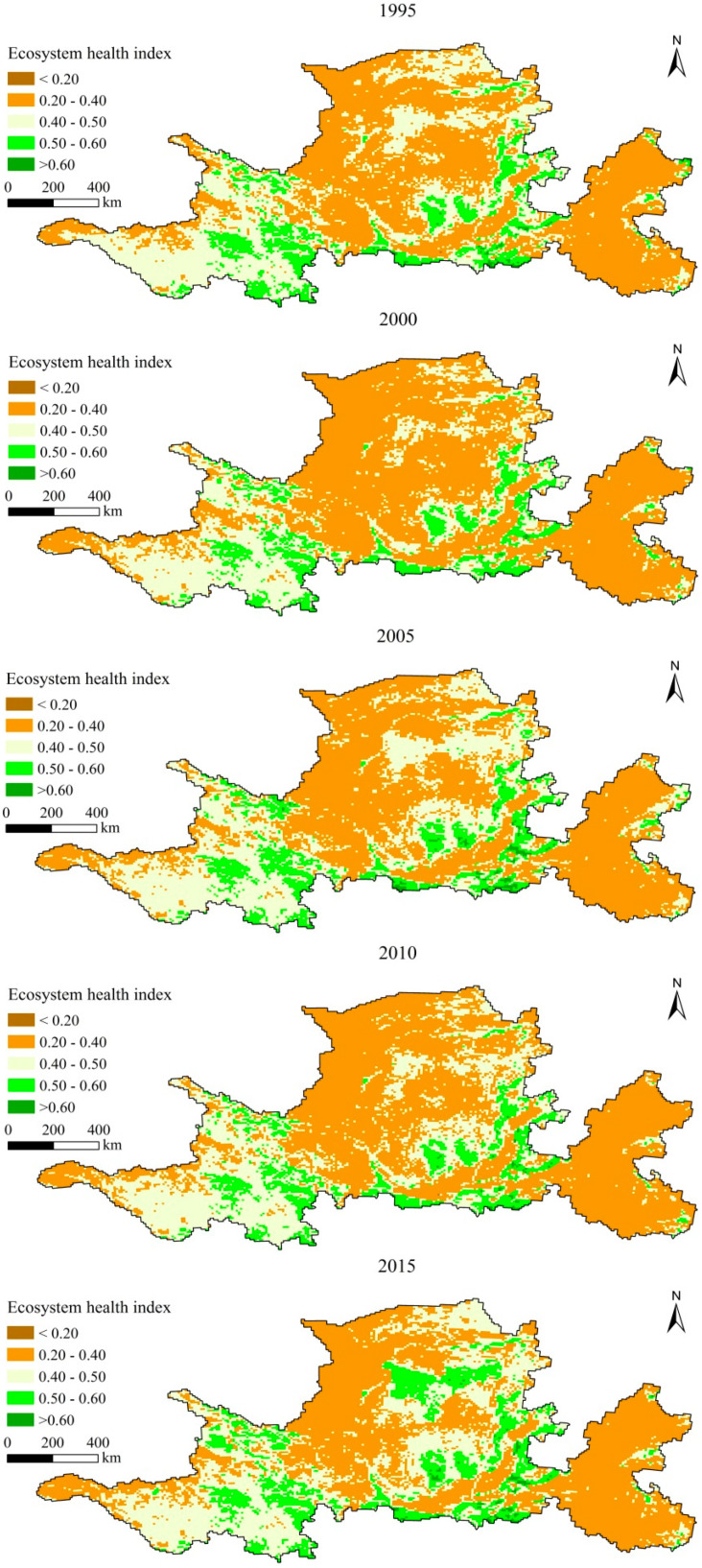
Spatial change of ecosystem health level in study area from 1995–2015.

The establishment of a comprehensive evaluation index system is one of the key steps in ecosystem health assessment. In the process, we comprehensively considered the following three aspects. First, each index was organized according to the PSR evaluation framework. The index system should fully reflect the service function of the ecosystem, as well as its complete structure and function, and it should also comprehensively and systematically represent the overall characteristics of the ecosystem. Second, the characteristics and internal differences of the study area were fully considered. Third, selection of specific indicators was based on their ability to be spatial, quantifiable, and operable. Using these three considerations, and on the basis of an extensive literature review, we constructed a comprehensive evaluation index system of ecosystem health, including resource pressure factor, social population pressure factor, vitality factor, organization factor, resilience factor, and natural system (Figure 2).

The pressure layer includes the resource pressure factor and population pressure factor. Land reclamation rate and per capita cultivated land area can reflect resource pressure [15,16,35,38]. The population density index and human disturbance index can reflect population pressure [16,35,39]. The Yellow River affected area is not only an important agricultural producing area in China, it is also an area of substantial population growth and industrial concentration. In order to reflect the resource pressure and social population pressure in the study area, we chose four indicators, including land reclamation rate, per capita cultivated land area, population density index and human disturbance index.

The state layer includes vigor factor, organization factor and resilience factor. Vigor represented ecosystem metabolism and productivity, organization assessed ecosystem diversity and interactions, and resilience measured capacity and resilience of maintaining ecosystem structure and function when facing interference [2]. The normalized difference vegetation index can reflect ecosystem metabolism and productivity [16], the biological abundance index can reflect ecosystem diversity and interaction [2,16], and the ecological resilience index can reflect the anti-interference ability and resilience of the ecosystem [2,16,20,40]. Therefore, the normalized difference vegetation index (NDVI), biological abundance index and ecological resilience index were used to reflect the vigor factor, organization factor, and resilience factor. Related studies have shown that ecosystem elasticity is reflected in two aspects [20]: (i) the ability to resist external interference, and (ii) the ability to restore the original state of the ecosystem after serious damage [39]. The degree of these two aspects is measured by the resistance and resilience coefficient, respectively. The calculation formula for the ecological resilience index is defined as [20,25]:

(1) $$\mathrm{RI}=0.6\times \overset{5}{\underset{i=1}{\sum }}P_{i}*Resist_{i}+0.4\times \overset{5}{\underset{i=1}{\sum }}P_{i}*Resil_{i}$$

In Formula (1), RI is the ecological resilience index; $P_{i}$ is the ratio of the area of the *i* landscape type to the area of the study unit; and $Resist_{i}$ and $Resil_{i}$ are the resistance coefficient and resilience coefficient of the *i* landscape type, respectively (Table 2). Xiao et al. point out that if the development level of the study area is relatively low, more attention should be paid to resistance [20]. Most of the areas in this study have a low level of economic development, so a higher weight value of 0.6 was used to emphasize resilience, and the weight of resistance was 0.4.

The response layer shows the response degree to the changes in ecosystem health conditions, including natural system factor and human activities factor [16,35]. Forest land coverage rate and wetland coverage rate indexes can reflect the response degree of the natural system [15,16,38]. Per capita GDP indicators can reflect the degree of response of human systems [16,38]. Therefore, forest land coverage rate, wetland coverage rate, and per capita GDP were used to reflect the natural system factor and human activities factor.

#### 3.2.3. Processing of Data and Determination of Indicator Weights

In order to eliminate possible differences in dimensions and orders of magnitude between indicators, we needed to standardize the index data. The maximum and minimum standardization method preserves the relationship existing in the original data and can eliminate the influence of dimension and data value range [16,20,26]. Therefore, it has become the most widely used standardized method in the field of ecosystem health research [16,20,26,28,40,41]. The standardized processing formulas of positive and negative indicators were:

(2) $$\{\begin{array}{c} X_{ij}^{p}=\frac{x_{ij}-MAX\left(x_{ij}\right)}{MAX\left(x_{ij}\right)-MIN\left(x_{ij}\right)}\;\\ X_{ij}^{n}=\frac{MAX\left(x_{ij}\right)-x_{ij}}{MAX\left(x_{ij}\right)-MIN\left(x_{ij}\right)} \end{array}$$

In Formula (2), $x_{ij}$ is the original index value; $x_{ij}^{p}$ and $x_{ij}^{n}$ are standardized values of positive and negative indicators, respectively; $\mathrm{MAX}\left(x_{ij}\right)$ is the maximum value of index *i* in year *j*; and $\mathrm{MIN}\left(x_{ij}\right)$ is the minimum value of indicator *i* in year *j*.

It was also important to determine the weight of the indicator. Considering that the index data of this paper is based on continuous raster data, we used the Delphi method and literature method to synthetically determine the index weight of criterion layer, factor layer and index layer [16]. Among them, the Delphi method was adopted to determine the weight of the indicator layer, and a number of scholars in the field of ecology and human geography were consulted by means of anonymous letter consultation, which was organized and statistically obtained (Table 3). The weight of criterion layer and factor layer referred to relevant research [14,15,16].

#### 3.2.4. Calculation of the Ecosystem Health Index

According to the PSR evaluation framework, we constructed a comprehensive evaluation model of ecosystem health, bringing the standardized index data and index weight into the evaluation model formula to calculate each criterion layer health index (PHI, SHI and RHI) and ecosystem health index (EHI). The specific calculation formula is as follows:

(3) $$\{\begin{array}{c} EHI=PHI*\omega_{P}+SHI*\omega_{S}+RHI*\omega_{R}\;\\ PHI=0.4\left(0.5*X_{1}+0.5*X_{2}\right)+0.6\left(0.5*X_{3}+0.5*X_{4}\right)\\ SHI=0.3*X_{5}+0.4*X_{6}+0.3*X_{7}\;\\ RHI=0.4\left(0.5*X_{8}+0.5*X_{9}\right)+0.6*X_{10}\; \end{array}\;$$

In Formula (3), *EHI* is the ecosystem health index; *PHI*, *SHI* and *RHI* are the health index of pressure, state and response layer, respectively; $\omega_{P}$,$\;\omega_{S}$ and $\omega_{R}$ are the weights of pressure, state and response layer, respectively; and $X_{i}$ is the standardized value of the *i* index.

### 3.3. Study of the Driving Factors of Ecosystem Health

#### 3.3.1. Factors Influencing Ecosystem Health

The operation mechanism of an ecosystem is complex, and the ecosystem health status is affected by natural factors and human factors [28]. We divided the influencing factors of ecosystem health into natural factors and man-made factors (Table 4). Natural factors included climate, topographic factors and geology, and resource endowments [16,28,38]; human factors were mainly related to human activities [38]. Two indicators of annual average temperature and annual average precipitation were selected to characterize climatic conditions; elevation and topographic fluctuation were selected to represent topographic conditions; soil type and soil erosion intensity were selected to characterize geological conditions; and net primary productivity (NPP) and biological abundance index were selected to represent resource endowment. The human interference index and population density were selected to represent the level of human activity.

The detailed calculation process for the influencing factors was: the four indicators of annual average temperature, annual average precipitation, elevation and NPP were used to obtain the average value of the research unit [28,38] using the regional statistical tools in ArcGIS10.3; the three indicators of topographic fluctuation, biological abundance index, human disturbance index and population density, were obtained by grid calculation. Soil type and soil erosion intensity were classified. With the help of ArcGIS10.3 software, the attribute value of the raster at the center point of the data raster layer was extracted by using the vector surface grid of 8 km rules [42].

For the calculation process of relief degree of land surface, refer to related research [43]. Relief degree of land surface (RDLS) is obtained by grid calculation. The specific calculation formula is as follows:

(4) RDLS={[Max(H)−Min(H)]×[1−P(A)/A]}/500

In Formula (4), RDLS is the relief degree of land surface, also known as the landform relief degree; *Max*(H) and *Min*(H) are the highest and lowest values of elevation in the region, respectively; P(A) is the area of flat land (km^2^); and A is the total area of the research unit (64 km^2^). In this study, we classified an area of land with slope less than or equal to 2° as flat land.

The calculation process for the biodiversity index, referring to the relevant research [2,44], is as follows:

(5) $$\mathrm{BI}=A_{\mathrm{bio}}\times \left(0.35\times A_{1}+0.21\times A_{2}+0.28\times A_{3}+0.11\times A_{4}+0.04\times A_{5}+0.01\times A_{6}\right)/A_{\mathrm{total}}$$

In Formula (5), BI is the biodiversity index; $A_{\mathrm{bio}}$ is the normalized coefficient of BI; $A_{1}$-$A_{6}$ is the area of forest land, grassland, water area, cultivated land, construction land and unused land; and $A_{\mathrm{total}}$ is the total area of the research unit.

Before using the Geodetector model for analysis, continuous data for all influencing factors needed to be discretized (classification processing) [42]. Average annual precipitation, annual average temperature, elevation, primary productivity (NPP), biological abundance index, human disturbance index and population density were divided into six categories and relief degree of land surface was divided into nine categories [38,45,46]. According to the relevant classification standards, soil type was divided into 16 categories and the soil erosion intensity was divided into six categories.

#### 3.3.2. Geodetector Model

The Geodetector method represents a new spatial statistics method that is used to detect spatial heterogeneity, and identify driving factors based on risk, ecology, and interaction [42]. This method overcomes the limitations of many assumptions and large amounts of data in the traditional mathematical statistical model.

(1) Detection of factors. The factor detector was used to analyze the factors affecting ecosystem health. The specific model formula is as follows:

(6) $$q=1-\left[\frac{\sum_{h=1}^{L}\sum_{i=1}^{N_{h}}{\left(Y_{hi}-\bar{Y_{h}}\right)}^{2}}{\sum_{i=1}^{N}{\left(Y_{i}-\bar{Y}\right)}^{2}}\right]=1-\frac{\sum_{h=1}^{L}N_{h}\sigma_{h}{}^{2}}{N\sigma^{2}}=1-\frac{SSW}{SST}$$

(7) $$SSW=\sum_{h=1}^{L}\sum_{i=1}^{N_{h}}{\left(Y_{hi}-\bar{Y_{h}}\right)}^{2}=\sum_{h=1}^{L}N_{h}\sigma_{h}{}^{2}$$

(8) $$SST=\sum_{i=1}^{N}{\left(Y_{i}-\bar{Y}\right)}^{2}=N\sigma^{2}$$

In Formula (6)–(8), *q* value represents the explanatory power of a factor on spatial differentiation of ecosystem health. *h* is the number of layers of the influencing factor; *N_h_* and *N* are the layer *h* of the influencing factor and the sample number of the whole study area, respectively; and $\sigma_{h}$ and σ are the variance of the ecosystem health index of layer *h* and the whole study area, respectively. $Y_{i}$ is the ecosystem health level in city *i*; $Y_{i}$ is the average of all the cities; $Y_{hi}$ is the ecosystem health level of city *i* in layer *h*; $Y_{h}$ is the average of all the cities in the *h* layer; *SSW* is the sum of in-layer variances; *SST* is the total variance of the study area. The range of *q* is [0, 1], and the higher the *q* value, the stronger is the explanatory power of this factor on ecosystem health level [42].

(2) Detection of factor interaction is used to identify the interaction between influencing factors, that is, to evaluate the accountability of the combined effect (enhancing or weakening) and their respective effect on the ecosystem health level.

(3) Detection of risk zones is used to judge whether there is a significant difference in mean attribute values between the subzones of two factors. The risk detection is examined by using T statistic value [42]:

(9) $$T=\frac{{\bar{Y}}_{h=1}-{\bar{Y}}_{h=2}}{{\left[\frac{Var\left(Y_{h=1}\right)}{n_{h=1}}+\frac{Var\left(Y_{h=2}\right)}{n_{h=2}}\right]}^{1/2}}$$

(4) For ecological detection, significant differences in the influence of any two factors on the spatial distribution of ecosystem health in study area were determined using the Geodetector method. The formula of the model is as follows [42]:

(10) $$F=\frac{N_{X_{1}}\left(N_{X_{2}}-1\right)\times SSW_{X_{1}}}{N_{X_{2}}\left(N_{X_{1}}-1\right)\times SSW_{X_{2}}}$$

(11) $$SSW_{X_{1}}=\overset{L_{1}}{\underset{h=1}{\sum }}N_{h}\sigma_{h}{}^{2}$$

(12) $$SSW_{X_{2}}=\overset{L_{2}}{\underset{h=1}{\sum }}N_{h}\sigma_{h}{}^{2}$$

In Formula (7)–(10), $N_{X_{1}}$ and $N_{X_{2}}$ denote the sample quantity of factor $X_{1}$ and $X_{2}$, respectively; $SSW_{X_{1}}$ and $SSW_{X_{2}}$ represent the sum of the intra-layer variances of the stratification formed by $X_{1}$ and $X_{2}$, respectively; and $L_{1}$ and $L_{2}$ is the number of layers of variables $X_{1}$ and $X_{2}$, respectively, where the zero hypothesis is H_0_: $SSW_{X_{1}}$= $SSW_{X_{2}}$. If *H_0_* was rejected at the significance level α, a significant difference in the influence of factors $X_{1}$ and $X_{2}$ on the spatial distribution of ecosystem health was determined.

## 4. Results

### 4.1. Spatiotemporal Evolution Characteristics of Ecosystem Health

#### 4.1.1. Spatial Patterns of Ecosystem Health Level

ArcGIS10.3 software was used to divide ecosystem health into five levels: lowest level (<0.20), low level (0.20–0.40), medium level (0.40–0.50), high level (0.50–0.60), and highest level (>0.60). From 1995 to 2015, low value areas of ecosystem health were mainly distributed in the Wulanbuhe Desert, the Tengger Desert and the Kubuqi Desert in the northwest of the study area, the Loess Plateau in the central areas, and the North China Plain in the east (Figure 2). Among these areas, the Wulanbuhe, Tengger and Kubuqi Desert had the lowest health level, followed by the North China Plain. The North China Plain was the area with the largest concentration of low-health units, due to this area being densely populated and an area of high industrial and agricultural production. Areas with a high level of ecosystem health were mainly distributed in Qinghai Lake and its surrounding areas in the northwest of the study area, ecological conservation areas in the upper reaches of the Yellow River, areas around the Taihang Mountains, the Qinling area, areas around Weishan Lake, and the Yellow River Delta. Among these, the ecological conservation area of the upper reaches of the Yellow River and the Qinling area recorded the highest health level, and the continuous area was larger.

#### 4.1.2. Spatial Change Trend Analysis of Ecosystem Health Level

**Figure 3:**
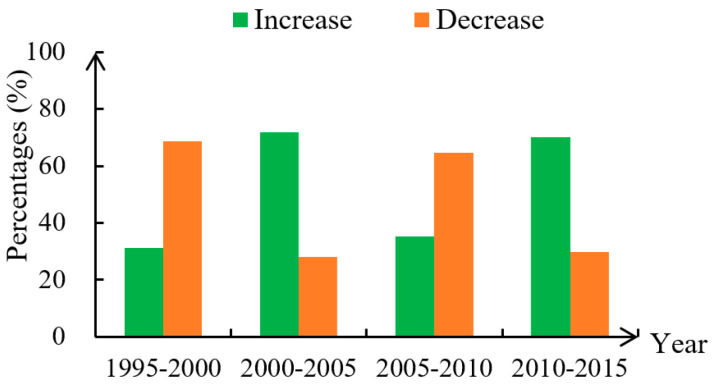
Changing trend of the proportion of increasing and decreasing units.

**Figure 4:**
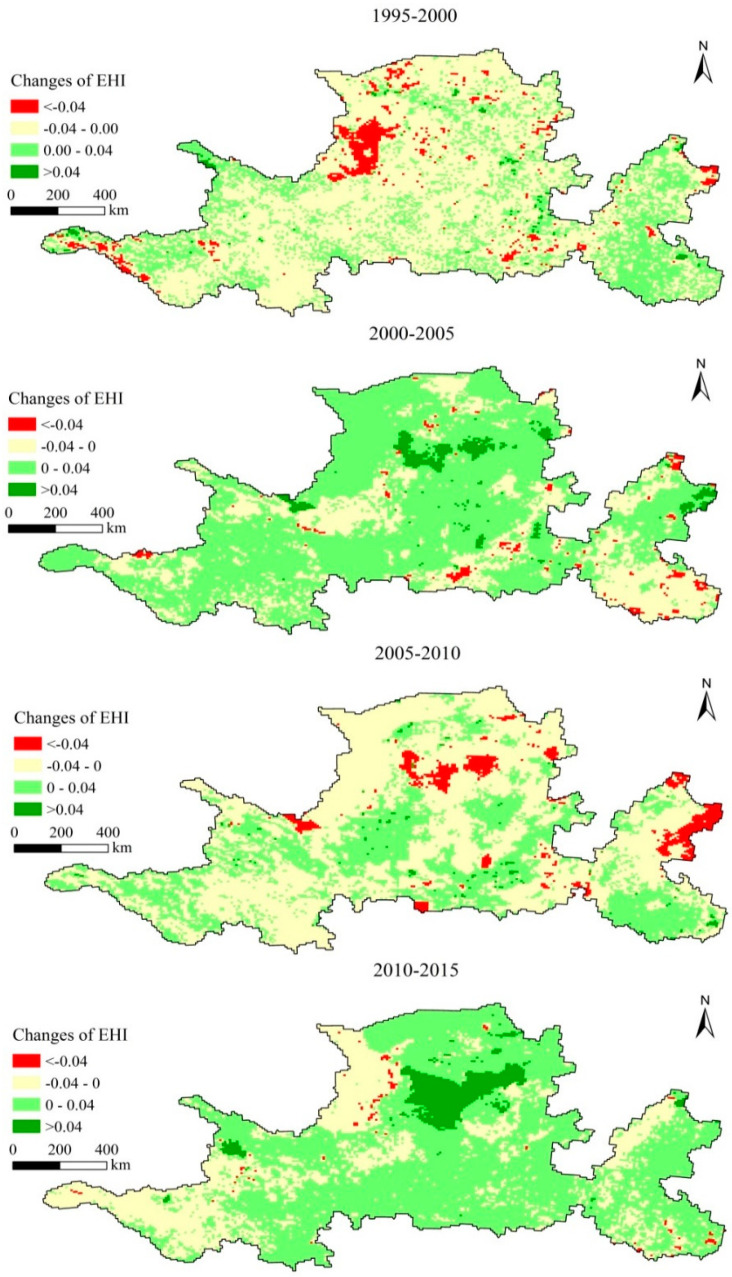
Changing trend of ecosystem health index from 1995 to 2015.

From 1995 to 2000, improved units of health level accounted for 31.24% of all units, while reduced units accounted for 68.76% (Figure 3), indicating that the overall health level of the study area had decreased. Results for spatial trends indicate that continuous areas with reduced levels of health were mainly distributed in the Wulanbuhe Desert, the Tengger Desert and the Kubuqi Desert, the Loess Plateau, the North China Plain, and the Yellow River Delta (Figure 4). In addition, cities and towns distributed across the study area and areas with a fragile ecological environment were also the main areas with reduced levels of health. Areas with great improvement in health were mainly distributed in areas around the Qilian Mountains in the northwest of the study area, which may be related to changes in regional temperature and precipitation.

From 2000 to 2005, improved units of health level accounted for 71.91% of all units, while reduced units accounted for 28.09% (Figure 3), indicating that the overall health level of the study area had improved. In detail, the areas with reduced levels of health were mainly distributed in the south central area of the North China Plain and the Beijing-Tianjin metropolitan region (Figure 4).The health level of the conservation area in the upper reaches of the Yellow River was recorded as improved.

From 2005 to 2010, improved units of health level accounted for 35.28% of all units, while reduced units accounted for 64.72%, indicating that the overall health level of the study area had significant decreased (Figure 3). Spatial trends during this period indicated health levels to have significantly decreased in the eastern and southern edge of the Tengger Desert, the eastern edge of the Kubuqi Desert, the central and northern part of the Loess Plateau, the West Henan Plain, the Beijing-Tianjin metropolitan area, and the Yellow River Delta (Figure 4).

From 2010 to 2015, improved units of health level accounted for 70.17% of all units, while reduced units accounted for 29.83%, indicating that the overall health level of the study area had significant improved (Figure 3). In detail, the health level of most units in the study area had improved, especially in the Qinghai Lake National Nature Reserve, the Loess Plateau, Central Inner Mongolia, Qinling Mountains, and the lower reaches of the Yellow River (Figure 4).

### 4.2. Influencing Factors on Ecosystem Health

#### 4.2.1. The Influence of the Detection Factor

The ecological detector of the Geodetector model was used to analyze whether there was a significant difference in the influence of any two factors on the spatial distribution of ecosystem health. The ecological detection results have shown that the differences among the factors were statistically significant (Table 5), indicating that the factors selected in the study were relatively reasonable. 

We used the factor detector of the Geodetector model to analyze the factors affecting the spatial differentiation of ecosystem health. In this way, the impact of various factors on ecosystem health were examined. Through the model calculation, the *q* value and *p* value of each factor were obtained (Table 6). In 2015, the effects of ten factors on ecosystem health can be arranged in the order of: biodiversity index (X8) > relief degree of land surface (X4) > soil type (X5) > annual mean precipitation (X2) > elevation (X3) > annual mean temperature (X1) > population density (X10) > soil erosion intensity (X6) > NPP (X7) > human disturbance index (X9).

The *q* value of the biological abundance index is the highest (0.783), indicating that this variable is the most important factor for determining the spatial pattern of ecosystem health (Table 6). Its power to explain the spatial pattern of ecosystem health reached 78.3%. Relief degree of land surface, soil type and average annual precipitation also have a very important impact on ecosystem health, with q values of 0.292, 0.253 and 0.250, respectively, and their explanatory power were 29.2%, 25.3% and 25%, respectively. The q values of elevation, annual average temperature and population density were all higher than 0.1, and their explanatory power were all greater than 10%. Although individual variables such as soil erosion intensity, NPP and human disturbance index have little effect on ecosystem health, these factors can be combined with other factors to have a significant impact on the spatial differentiation pattern of ecosystem health.

#### 4.2.2. Indicative Effect Analysis

In our study, the risk detector of the Geodetector model was used to analyze the appropriate type or range of influencing factors (Table 7), and a statistical significance test with a confidence level of 95% was undertaken. The more suitable the type or range of detection factors are, the more beneficial to ecosystem health.

Ecosystem health level was the highest when annual average temperature of the year was 0.04–3.67 °C and average annual precipitation was 350.78–494.52 mm (Table 7). When elevation was 2851.18–3953.34 m and the relief degree of land surface was 1.48–1.92, ecosystem health improvement was most marked. Ecosystem health was found to be conducive to improvement when soil type was leaching soil and soil erosion intensity was the mild erosion type. Ecosystem health was also likely to be improved when NPP was 253.68–428.77 and the biological abundance index was 0.27–0.49 (the highest). When the human disturbance index is 0–0.03 (the lowest) and the population density was 0.06–153.53 (the lowest), ecosystem health level is the highest, indicating that human disturbance index and population density have a negative impact on ecosystem health.

#### 4.2.3. Analysis of the Interaction between Factors

**Figure 5:**
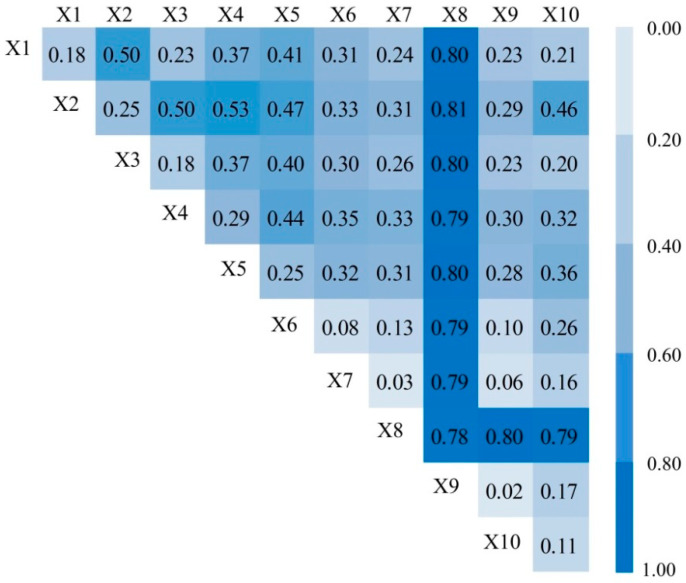
Interaction detection of factors.

The interaction detector can identify the interaction between different detection factors, indicating whether the joint action of evaluation factors A and B will increase or weaken the explanatory power of the dependent variable Y, or whether the effects of these factors on the dependent variable are independent of each other. The *q* values of (1,1), (2,2), (3,3) and other positions in the table represent the effect of a single factor on ecosystem health, and the *q* values of other locations indicate the interaction level of the superposition of the two factors on ecosystem health (Figure 5). The interaction between factors can be divided into weakening, mutual enhancement, and nonlinear enhancement.

The explanatory power (*q* value) of the interaction between factors on ecosystem health was higher than that of any single factor on ecosystem health (Figure 5), indicating that the interaction between the detection factors on ecosystem health was significantly enhanced. From the improvement level of the *q* value, the superposition of X1∩X2, X1∩X6, X1∩X7, X2∩X3, X2∩X4, X2∩X5, X2∩X6, X2∩X9, X2∩X10, X3∩X5, X3∩X6, X3∩X7, X4∩X5, X5∩X7 and X6∩X10 had a significant interactive effect on ecosystem health.

From the point of view of the type of interaction, the interaction between factors showed mutual and non-linear enhancement (Table 8). The proportion of the two interaction types was 55.6% and 44.4%, respectively. We compared the PD value of the interaction between the two factors with the sum of the *q* values of the two factors, and found that the PD value of 44.4% of the factor combinations was greater than the sum of the *q* values of the two factors, indicating that the interaction of most of the factor combinations greatly enhanced the impact on ecosystem health. In conclusion, the effects of natural factors and human factors on ecosystem health are not independent of each other but interact with each other. The interaction of multiple factors on ecosystem health is thus not a simple superposition process, rather a mutual or non-linear enhancement.

## 5. Discussion

### 5.1. Comparison with Previous Studies

Our results show that ecosystem health presents significant spatial differences in the study area. The ecosystem health level of Wulanbuhe Desert, Tengger Desert and Kubuqi Desert in the northwest of the study area is the lowest. This was in line with the study of He et al. (2019) [33]. The ecosystem health level in the three desert fringe areas decreased before 2010, but gradually improved after 2010 (Figure 3). This result has been verified by Bai et al. (2020) [47]. The main reason is that the ecosystem health level of the desert edge area has decreased due to the influence of land desertification before 2010. Thanks to China’s large-scale desert control project, desertification has been effectively curbed since 2010 (after nearly 30 years of efforts, the Kubuqi Desert in the western part of China’s Inner Mongolia Autonomous region has become the first desert in the world to be completely managed in this way). The north China Plain has a low level of ecosystem health due to frequent human activities. Song et al. (2015) [48] found that socio-economic activities in the North China Plain lead to significant changes in land use types, which leads to the decline of ecosystem services and ecosystem health, as in our own research results. The ecosystem health level of the Yellow River Delta and the Loess Plateau is low and fluctuates greatly. This is consistent with the relevant research results [16,28]. The Yellow River Delta and the Loess Plateau are ecologically fragile areas. Land cover change, soil erosion and environmental pollution directly lead to the decline of ecosystem health. Fluctuation changes during the study period are mainly related to regional ecological protection policies [33].

The results of influencing factors show that different factors have different explanatory power to explain the spatial differentiation of ecosystem health. Biodiversity index, relief degree of land surface and soil type are the main factors affecting the spatial differentiation of ecosystem health. The interaction of relief degree of land surface with annual average precipitation and soil erosion intensity enhanced the explanatory power of relief degree of land surface (Figure 5), indicating that the superposition of climatic, topographic and soil erosion factors significantly affected the spatial differentiation of ecosystem health. For example, the combination of topography and climate causes serious soil erosion on the Loess Plateau, which then has a negative impact on regional ecosystem health. The interaction of annual average precipitation, relief degree of land surface, soil erosion and population density enhanced the single factor explanatory power on spatial differentiation of ecosystem health (Figure 5). This indicates that the interaction of climatic factors (precipitation), topographical factors (topographical fluctuations, soil erosion) and human activity factors can significantly enhance the impact on ecosystem health. Similar studies have shown that interaction between moisture indexes and land use intensity enhances the explanatory power of moisture indexes on regional differences [33]. It can be seen that human activities play a catalytic role in enhancing the influence of natural environmental factors. For example, in ecologically fragile regions such as Inner Mongolia grassland, Loess Plateau and Yellow River Delta, human activities combined with adverse natural factors can significantly reduce the health level of the regional ecosystem.

### 5.2. Implications for Ecological Conservation

Geospatial information knowledge mining can not only reveal the temporal and spatial distribution and agglomeration characteristics of ecosystem health, but also find the spatial risk factors that have an important impact and identify the hotspots for ecological risk. This study can provide important suggestions for the formulation of differential ecological protection and restoration measures [20].

Our research results identified the spatial differences and driving factors of ecosystem health, so we can take targeted ecological protection measures according to the situation in different regions. For example, in the Ulaanbuhe, Tengger and Kubuzi deserts with the lowest level of ecosystem health, engineering measures to prevent the movement of sand dunes are needed to prevent desert expansion [47]; in addition, surface vegetation can be restored by planting a large number of drought-resistant plants. In the Loess Plateau, vegetation coverage can be increased by plants with developed roots and strong soil fixation capacity, so as to slow down soil and water loss [16,33]. In the Yellow River Delta, it is necessary to draw a red line for ecological protection to prohibit the destruction of wetlands and to build coastal dams to prevent the backflow of sea water [28,33]. In the North China Plain, the destruction of land cover can be reduced by reasonably controlling the speed of urban expansion and optimizing land use structure, while optimizing industrial structure with less pollutant emissions [48,49].

### 5.3. Limitations and Future Work

Determining index weight is also an important part of ecosystem health evaluation. There is no doubt that the index weight will have an important impact on the final result, so its setting is extremely important. In previous studies, part of the research used objective weight determination methods to determine indicator weight, including the analytic hierarchy process [25,50], entropy weight method [20,22,23], mean square error method and TOPSIS (technique for order preference by similarity to an ideal solution) model [23,51]. Other studies used subjective weight determination, including the Delphi method [16] and empirical method [20,36]. From a dialectical point of view, objective weight determination and subjective weight determination methods have their own advantages and disadvantages which are suitable for different studies. In this study, considering that the index data in this paper was based on continuous raster data, this paper adopts the Delphi method [16] and literature method [14,15] to comprehensively determine the indicator weight of the criterion layer, factor layer and index layer. Although this method has a certain subjectivity, it highlights the relative importance of the evaluation index in combination with the actual situation and takes the knowledge and experience of experts and professionals into account. Not only that, but the ecosystem health assessment itself is also a judgment driven by human subjective values [20].

In this study, the “stress-state-response” (PSR) evaluation framework model was selected to organize evaluation indicators, so as to comprehensively evaluate the health status of the ecosystem in the study area. However, as the relationship between natural ecosystem and socio-economic system is still weak, the improvement of the framework still needs further study. Based on the assessment framework, we establish a comprehensive evaluation index system using multi-source data. Compared with other studies [2,20,41], the indicator system of this paper covered the natural, social, and economic aspects of the ecosystem, therefore making evaluation results more accurate. However, due to accessibility, representativeness and objectivity of the data, there are some limitations in using various indicators to comprehensively evaluate the level of ecosystem health. In order to improve the accuracy of ecosystem health assessment, future investigations need to expand in using more data sources and index types. There is no doubt that although the evaluation model and index system of this study were not the most ideal, they were very suitable for regional ecosystem health assessment with different climate types, landforms, and development level.

In terms of influencing factors, although our research has provided important information for future research, two areas require further analysis: firstly, due to difficulties in obtaining data, influencing factors selected in our study may not be comprehensive enough, and research data used in our study presents certain issues, such as having a large resolution and only covering a short time period. This issue can be addressed by expanding the use of more types of factor and improving the accuracy and time span of the research data. Secondly, as extremely complex interactions existed among the influencing factors on ecosystem health [42], so clarifying the complex interaction mechanism among factors plays an important role in further detailed study of ecosystem health.

## 6. Conclusions

In this study, we constructed an extended evaluation index system and a comprehensive evaluation model based on the pressure-state-response (PSR) framework and used RS and GIS techniques to dynamically evaluate the ecosystem health level in the Yellow River affected area. Influencing factors on ecosystem health spatial differentiation were quantitatively analyzed using the Geodetector model. The main findings are:

From 1995 to 2015, the ecosystem health level in the Yellow River affected area showed obvious characteristics of spatial differentiation. From the perspective of the interannual variation trend of ecosystem health level, the overall ecosystem health level from 1995 to 2005 presented a trend first of decline and then of rise, and the same trend occurred from 2005 to 2015. Areas with large fluctuations in health level were mainly distributed in extreme climate areas (deserts), ecologically fragile areas (the Loess Plateau, arid grasslands), and plain and hilly areas (the North China Plain).

Biodiversity index, relief degree of land surface, soil type, annual average precipitation, elevation, annual average temperature, and population density have strong explanatory power for the spatial differentiation of ecosystem health in the study area. Interaction analysis showed that obvious interactions between influencing factors existed, indicating that the effects of natural factors and human factors on ecosystem health are not independent of each other, but interact with each other. The interaction of natural factors such as climate, topography and soil erosion significantly enhances the impact on ecosystem health. The interaction of climatic factors (precipitation), topographical factors (relief degree of land surface, soil erosion) and human activity factors can significantly enhance the impact on ecosystem health. Therefore, human activity factors play a catalytic role in enhancing the influence of natural environmental factors, especially in ecologically fragile regions.

## Figures and Tables

**Table 1:** Environmental and socio-economic variables used in the study.

Variable Name	Data Type	Resolution	Unit	Source
Study area boundary	Vector data	-	-	http://www.geodata.cn
Land use data	Raster data	1 km	-	http://www.resdc.cn
Normalized difference vegetation index	Raster data	1 km	-	http://www.resdc.cn
Vegetation net primary productivity	Raster data	1 km	Gram carbon/m^2^	http://www.dsac.cn
Annual average temperature	Raster data	1 km	mm	http://www.resdc.cn
Annual average precipitation	Raster data	1 km	°C	http://www.resdc.cn
Elevation	Raster data	1 km	Meter	http://www.resdc.cn
Slope	Raster data	1 km	Degree	http://www.resdc.cn
Soil type	Raster data	1 km	-	http://www.resdc.cn
Soil erosion intensity	Raster data	1 km	t/(km^2^ × a)	http://www.resdc.cn
GDP spatial distribution date	Raster data	1 km	Yuan	http://www.resdc.cn
Population spatial distribution date	Raster data	1 km	Person	http://www.resdc.cn

**Table 2:** The ecosystem resistance coefficient and resilience coefficient of each landscape type.

Landscape Type	Forest Land	Grassland	Farmland	Construction Land	Water Body
Resistance	1	0.6	0.5	0.3	0.8
Resilience	0.6	0.8	0.3	0.2	0.7

**Table 3:** Index system of ecosystem health assessment.

Object	Criteria Layer		Elements Layer		Indicators Layer	
	Criteria	Weight	Elements	Weight	Indicators	Weight
Ecosystem	Pressure	0.3	Resource pressure	0.4	Land reclamation rate	0.5
					Per capita cultivated land area	0.5
			Population pressure	0.6	Population density index	0.5
					Human disturbance index	0.5
health	State	0.4	Vitality	0.3	NDVI	1
			Organization	0.4	Biological abundance index	1
			Resilience	0.3	Ecological elasticity index	1
	Response	0.3	Natural system	0.4	Forest land coverage rate	0.5
					Wetland coverage rate	0.5
			Human activities	0.6	Per capita GDP	1

**Table 4:** Index system of influencing factors.

Influencing Factor	Factors	Code	Indicators
Natural factors	Climatic conditions	X1	Annual mean temperature
		X2	Annual mean precipitation
	Topographic and geological conditions	X3	Elevation
		X4	Relief degree of land surface
		X5	Soil type
		X6	Soil erosion intensity
	Resource endowments	X7	NPP
		X8	Biodiversity index
Human factors	Human activities	X9	Human disturbance index
		X10	Population density

**Table 5:** Statistical significance of detection factors (95% confidence level).

Factors	X1	X2	X3	X4	X5	X6	X7	X8	X9	X10
X1										
X2	Y									
X3	N	Y								
X4	Y	Y	Y							
X5	Y	N	Y	Y						
X6	Y	Y	Y	Y	Y					
X7	Y	Y	Y	Y	Y	Y				
X8	Y	Y	Y	Y	Y	Y	Y			
X9	Y	Y	Y	Y	Y	Y	N	Y		
X10	Y	Y	Y	Y	Y	Y	Y	Y	Y	

**Table 6:** *q* statistic and *p* values of detection factors in 2015.

	X1	X2	X3	X4	X5	X6	X7	X8	X9	X10
*q* statistic	0.176	0.250	0.180	0.292	0.253	0.080	0.034	0.783	0.023	0.110
*p* value	0.000	0.000	0.000	0.000	0.000	0.000	0.000	0.000	0.000	0.000

**Table 7:** Suitable limits of natural factors (95% confidence level).

Code	Indicators	Suitable Types or Range
X1	Annual mean temperature	0.04–3.67
X2	Annual mean precipitation	350.78–494.52
X3	Elevation	2851.18–3953.34
X4	Relief degree of land surface	1.48–1.92
X5	Soil type	Leaching soil
X6	Soil erosion intensity	Mild erosion
X7	NPP	253.68–428.77
X8	Biological abundance index	0.27–0.49
X9	Human disturbance index	0–0.03
X10	Population density	0.06–153.53

**Table 8:** Interaction detection between ecosystem health influence factors.

A∩B	A+B	Interpretation	A∩B	A+B	Interpretation
X1∩X2 = 0.500	>0.426 = X1∩X2	↑	X3∩X10 = 0.204	<0.290 = X3∩X10	↑↑
X1∩X3 = 0231	<0.356 = X1∩X3	↑↑	X4∩X5 = 0.437	<0.545 = X4∩X5	↑↑
X1∩X4 = 0.371	<0.468 = X1∩X4	↑↑	X4∩X6 = 0.350	<0.372 = X4∩X6	↑↑
X1∩X5 = 0.408	<0.429 = X1∩X5	↑↑	X4∩X7 = 0.334	>0.326 = X4∩X7	↑
X1∩X6 = 0.308	>0.256 = X1∩X6	↑	X4∩X8 = 0.792	<1.075 = X4∩X8	↑↑
X1∩X7 = 0.243	>0.210 = X1∩X7	↑	X4∩X9 = 0.303	<0.315 = X4∩X9	↑↑
X1∩X8 = 0.802	<0.959 = X1∩X8	↑↑	X4∩X10 = 0.324	<0.402 = X4∩X10	↑↑
X1∩X9 = 0.226	>0.199 = X1∩X9	↑	X5∩X6 = 0.323	<0.333 = X5∩X6	↑↑
X1∩X10 = 0.205	<0.286 = X1∩X10	↑↑	X5∩X7 = 0.305	>0.287 = X5∩X7	↑
X2∩X3 = 0.499	>0.429 = X2∩X3	↑	X5∩X8 = 0.802	<1.036 = X5∩X8	↑↑
X2∩X4=0.525	<0.542 = X2∩X4	↑↑	X5∩X9 = 0.283	>0.276 = X5∩X9	↑
X2∩X5 = 0.474	<0.503 = X2∩X5	↑↑	X5∩X10 = 0.356	<0.363 = X5∩X10	↑↑
X2∩X6 = 0.333	>0.329 = X2∩X6	↑	X6∩X7 = 0.130	>0.113 = X6∩X7	↑
X2∩X7 = 0.306	>0.286 = X2∩X7	↑	X6∩X8 = 0.789	<0.863 = X6∩X8	↑↑
X2∩X8 = 0.814	<1.033 = X2∩X8	↑↑	X6∩X9 = 0.101	<0.103 = X6∩X9	↑↑
X2∩X9 = 0.292	>0.273 = X2∩X9	↑	X6∩X10 = 0.255	>0.190 = X6∩X10	↑
X2∩X10 = 0.456	>0.360 = X2∩X10	↑	X7∩X8 = 0.787	<0.817 = X7∩X8	↑↑
X3∩X4 = 0.371	<0.472 = X3∩X4	↑↑	X7∩X9 = 0.058	>0.057 = X7∩X9	↑
X3∩X5 = 0.399	<0.433 = X3∩X5	↑↑	X7∩X10 = 0.161	>0.144 = X7∩X10	↑
X3∩X6 = 0.302	>0.259 = X3∩X6	↑	X8∩X9 = 0.801	<0.806 = X8∩X9	↑↑
X3∩X7 = 0.259	>0.213 = X3∩X7	↑	X8∩X10 = 0.791	<0.893 = X8∩X10	↑↑
X3∩X8 = 0.803	<0.963 = X3∩X8	↑↑	X9∩X10 = 0.166	>0.133 = X9∩X10	↑
X3∩X9 = 0.226	>0.203 = X3∩X9	↑			

Note: “↑↑”denotes A and B enhance each other; “↑”denotes a non-linear enhancement of A and B.

## References

[1] Chen D.L., Lu X.H., Liu X., Wang X. (2019). Measurement of the eco-environmental effects of urban sprawl: Theoretical mechanism and spatiotemporal differentiation. Ecol. Indic..

[2] Yan Y., Zhao C.L., Wang C.X., Shan P., Zhang Y.J., Wu G. (2016). Ecosystem health assessment of the Liao River Basin upstream region based on ecosystem services. Acta Ecol. Sin..

[3] Zhang H., Qi Z.F., Ye X.Y., Cai Y.B., Ma W.C., Chen M.N. (2013). Analysis of land use/land cover change, population shift, and their effects on spatiotemporal patterns of urban heat islands in metropolitan Shanghai, China. Appl. Geogr..

[4] Lu L.L., Guo H.D., Corbane C., Li Q.T. (2019). Urban sprawl in provincial capital cities in China: Evidence from multi-temporal urban land products using Landsat data. Sci. Bull..

[5] Peng J.F., Peng K.Y., Li J.B. (2018). Climate-Growth response of Chinese white pine (Pinus armandii) at different age groups in the Baiyunshan National Nature Reserve, central China. Dendrochronologia.

[6] Xia H.M., Qin Y.C., Feng G., Meng Q.M., Cui Y.P., Song H.Q., Ouyang Y., Liu G.J. (2019). Forest phenology dynamics to climate change and topography in a geographic and climate transition zone: The Qinling Mountains in central China. Forests.

[7] Wu H., Ding J.Q. (2019). Global Change Sharpens the Double-Edged Sword Effect of Aquatic Alien Plants in China and Beyond. Plant Sci..

[8] Rapport D.J., Riegier H.A., Hutchinson T.C. (1985). Ecosystem behavior under stress. Am. Nat..

[9] Shear H. (1996). The development and use of indicators to assess the state of ecosystem health in the Great Lakes. Ecosyst. Health.

[10] Lepold J.C. (1997). Getting a handle on ecosystem health. Science.

[11] Rapport D.J. (1989). What constitutes ecosystem health?. Perspect. Biol. Med..

[12] Rapport D.J. (2011). Eco-Cultural health, global health, and sustainability. Ecol. Res..

[13] Pan Y., Xu Z.R., Yu C.Q., Tu Y.L., Li Y., Wu J.X. (2013). Spatiotemporal variation of interacting relationships among multiple provisioning and regulating services of Tibet grassland ecosystem. Acta Ecol. Sin..

[14] Ou C.H., Liu W.H. (2010). Developing a sustainable indicator system based on the pressure-state-response framework for local fisheries: A case study of Gungliau, Taiwan. Ocean Coast. Manag..

[15] Sun T.T., Lin W.P., Chen G.S., Guo P.P., Zeng Y. (2016). Wetland ecosystem health assessment through integrating remote sensing and inventory data with an assessment model for the Hangzhou Bay, China. Sci. Total Environ..

[16] Liu D.L., Hao S.L. (2017). Ecosystem health assessment at county-scale using the pressure-state-response framework on the Loess Plateau, China. Int. J. Environ. Res. Public Health.

[17] Costanza R., Norton B.G., Haskell B.D. (1992). Ecosystem Health: New Goals for Environmental Management.

[18] Lu F., Li Z. (2003). A model of ecosystem health and its application. Ecol. Model..

[19] Chaves H.M.L., Alipaz S. (2007). An integrated indicator based on basin hydrology, environment, life, and policy: The watershed sustainability index. Water Res. Manag..

[20] Xiao R., Liu Y., Fei X.F., Yu W.X., Zhang Z.H., Meng Q.X. (2019). Ecosystem health assessment: A comprehensive and detailed analysis of the case study in coastal metropolitan region, eastern China. Ecol. Indic..

[21] Zeng C., Deng X.Z., Xu S., Wang Y.T., Cui J.X. (2016). An integrated approach for assessing the urban ecosystem health of megacities in China. Cities.

[22] Bebianno M.J., Pereira C.G., Rey F., Cravo A., Duarte D., D’Errico G., Regoli F. (2015). Integrated approach to assess ecosystem health in harbor areas. Sci. Total Environ..

[23] Meng L.R., Huang J., Dong J.H. (2018). Assessment of rural ecosystem health and type classification in Jiangsu province, China. Sci. Total Environ..

[24] Hong H.K., Liao H.P., Wei C.F., Li T., Xie D.T. (2015). Health assessment of a land use system used in the ecologically sensitive area of the Three Gorges reservoir area, based on the improved TOPSIS Method. Acta Ecol. Sin..

[25] Peng J., Liu Y.X., Li T.Y., Wu J.S. (2017). Regional ecosystem health response to rural land use change: A case study in Lijiang City, China. Ecol. Indic..

[26] Shen C.C., Shi H.H., Zheng W., Ding D.W. (2016). Spatial heterogeneity of ecosystem health and its sensitivity to pressure in the waters of nearshore archipelago. Ecol. Indic..

[27] Wang G.C., Li P.F., Wang H., Li G.J. Assessments of integrated ecosystems in mining areas based on energy analysis. Acta Ecol. Sin..

[28] Chi Y., Zheng W., Shi H.H., Sun J.K., Fu Z.Y. (2018). Spatial heterogeneity of estuarine wetland ecosystem health influenced by complex natural and anthropogenic factors. Sci. Total Environ..

[29] Bae D.Y., Kumar H.K., Han J.H., Kim J.Y., Kim K.W., Kwon Y.H., An K.G. (2010). Integrative ecological health assessments of an acid mine stream and in situ pilot tests for wastewater treatments. Ecol. Eng..

[30] Cheng X., Chen L.D., Sun R.H., Kong P.R. (2018). Land use changes and socio-economic development strongly deteriorate river ecosystem health in one of the largest basins in China. Sci. Total Environ..

[31] Ye C., Li C.H., Wang Q.G., Chen X.G. (2012). Driving forces analysis for ecosystem health status of littoral zone with dikes: A case study of Lake Taihu. Acta Ecol. Sin..

[32] Xie G.D., Zhang C.X., Zhen L., Zhang L.M. (2017). Dynamic changes in the value of China’s ecosystem services. Ecosyst. Serv..

[33] He J.H., Pan Z.Z., Liu D.F., Guo X.N. (2019). Exploring the regional differences of ecosystem health and its driving factors in China. Sci. Total Environ..

[34] Santos J.E., Nogueira F., Pires J.S., Obara A.T., Pires A.M. (2001). The value of the Ecological Station of Jataí’s ecosystem services and natural capital. Braz. J. Biol..

[35] Xu M.D., Li J., Peng J., Niu J., Cao L. (2010). Ecosystem health assessment based on RS and GIS. Ecol. Environ. Sci..

[36] Chi Y., Shi H.H., Zheng W., Sun J.K., Fu Z.Y. (2018). Spatiotemporal characteristics and ecological effects of the human interference index of the Yellow River Delta in the last 30 years. Ecol. Indic..

[37] Ji X.P., Bai Y.P., Du H.B., Wang J.B., Zhou L. (2017). Research on the spatial quantitative evaluation and coupling coordination degree of ecological carrying capacity in Gansu Province. Acta Ecol. Sin..

[38] Yao P.P., Wang W., Sun R., Yue B., Liu G. (2018). Ecosystem Health Assessment of Wetlands in Yangtze River Basin. Met. Environ. Sci..

[39] Chang Y., Hou K., Wu Y.P., Li X.X., Zhang J.L. (2019). A conceptual framework for establishing the index system of ecological environment evaluation–A case study of the upper Hanjiang River, China. Ecol. Indic..

[40] Kang P., Chen W.P., Hou Y., Li Y.Z. (2018). Linking ecosystem services and ecosystem health to ecological risk assessment: A case study of the Beijing-Tianjin-Hebei urban agglomeration. Sci. Total Environ..

[41] Xia H.M., Zhao J., Qin Y.C., Yang J., Cui Y.P., Song H.Q., Ma L.Q., Jin N., Meng Q.M. (2019). Changes in Water Surface Area during 1989–2017 in the Huai River Basin using Landsat Data and Google Earth Engine. Remote Sens..

[42] Wang J.F., Xu C.D. (2017). Geodetector: Principle and prospective. Acta Geogr. Sin..

[43] Zhang J.J., Zhu W.B., Zhu L.Q., Cui Y.P., He S.S., Ren H. (2019). Spatial variation of terrain relief and its impacts on population and economy based on raster data in West Henan Mountain Area. J. Geogr. Sci..

[44] Yao Y., Wang S.X., Zhou Y., Liu R., Han X.D. (2012). The application of ecological environment index model on the national evaluation of ecological environment quality. Remote Sens. Inf..

[45] Liu Y.S., Li J.T. (2017). Geographic detection and optimizing decision of the differentiation mechanism of rural poverty in China. Acta Geogr. Sin..

[46] Peng W.F., Kuang T.T., Tao S. (2019). Quantifying influences of natural factors on vegetation NDVI changes based on geographical detector in Sichuan, western China. J. Clean. Prod..

[47] Bai X.L., Wang L.X., Ji S.X., Chen Z.X., Chang X.L. (2020). Assessment of ecosystem health in grassland-desert ecotone in northern Ordos: A case study of Ten Tributaries Basin. J. Desert Res..

[48] Song W., Deng X.Z., Yuan Y.W., Wang Z., Li Z.H. (2015). Impacts of land-use change on valued ecosystem service in rapidly urbanized North China Plain. Ecol. Model..

[49] Luo Q.L., Zhuo J.F., Li Z.G., Yu B.L. (2020). Spatial differences of ecosystem services and their driving factors: A comparation analysis among three urban agglomerations in China’s Yangtze River Economic Belt. Sci. Total Environ..

[50] Sun B.D., Tang J.C., Yu D.H., Song Z.W., Wang P.G. (2019). Ecosystem health assessment: A PSR analysis combining AHP and FCE methods for Jiaozhou Bay, China. Ocean Coast. Manag..

[51] Turner M.G. (1989). Landscape ecology: The effect of pattern on process. Annu. Rev. Ecol. Syst..

